# The Cost of Becoming a Surgeon: A Comparative Analysis of Human and Veterinary Surgical Specialisation in the United Kingdom

**DOI:** 10.7759/cureus.96921

**Published:** 2025-11-15

**Authors:** Matthew How Saw Keng, Megan Anley

**Affiliations:** 1 Trauma and Orthopaedics, Derriford Hospital, Plymouth, GBR; 2 General Practice, Woodlands Veterinary Hospital, Plymouth, GBR

**Keywords:** burnout, career choice, economics of education, education, medical, postgraduate education, specialization, surgery, veterinary, workforce retention

## Abstract

Background

Medicine and veterinary science share similar undergraduate foundations yet diverge significantly after qualification. Medical graduates progress through salaried, structured NHS training, whereas veterinarians enter privately funded, variable pathways toward specialist recognition.

Aim

The main aim of this study is to compare duration, cost, salary trajectory, burnout, and retention across human and veterinary surgical specialisation in the United Kingdom, using a cross-sectional comparative design.

Methods

Cross-sectional descriptive comparative study using 2024-2025 publicly available UK data from regulatory, educational, and professional bodies. Domains included training duration, financial cost, salary progression, burnout, and retention.

Results

Human surgical training averages 15 years versus 11-12 for veterinary surgeons. Estimated personal costs totalled £48,600 (human) and £56,000 (veterinary). Human trainees are salaried throughout (£38,000-£145,000), whereas veterinary trainees earn £19,600-£51,000 during internships and residencies before reaching £70,000-£100,000+. Reported burnout prevalence ranged from approximately 52% (veterinary) to 66% (medical), based on profession-specific national surveys rather than directly comparable instruments. Retention exceeded 70% at 10 years for human consultants; precise veterinary specialist data were unavailable. Salaries vary by institution and are subject to change; the figures reported reflect data available at the time of the search.

Conclusion

Veterinary surgical training is shorter but financially riskier, with delayed return on investment and limited structural support. Both professions face high burnout and retention pressures, underscoring the need for sustainable, well-supported postgraduate frameworks.

## Introduction

Undergraduate degrees in medicine and veterinary science share a strong scientific foundation and similar pedagogical design, including problem-based learning, anatomy dissection, and clinical rotations. After graduation, doctors enter a nationally coordinated NHS training pathway leading to Fellowship of the Royal College of Surgeons (FRCS) and a Certificate of Completion of Training (CCT) [1].

In contrast, veterinary graduates usually work in general practice before pursuing competitive internships and residencies accredited by the European College of Veterinary Surgeons (ECVS) or the Royal College of Veterinary Surgeons (RCVS) [2,3]. While both professions demand high technical proficiency and responsibility, their postgraduate structures differ in regulation, funding, and financial security.

Previous research has highlighted high burnout and psychological strain among both medical [4-8] and veterinary professionals [9-13]. However, no UK-based comparative analysis has examined training duration, cost, remuneration, and well-being outcomes between the two professions.

To date, no published work has systematically compared the pathways, costs, and professional outcomes of surgical specialisation between human and veterinary medicine in the United Kingdom. The existing literature in both sectors addresses burnout or workforce trends in isolation, but does not cross-link these data [4-13]. Consequently, this study represents the first descriptive comparison of its kind, and findings should be interpreted in that context.

Therefore, the objectives of this study were to conduct a cross-sectional comparative analysis of (1) training structure, (2) financial cost, (3) salary progression, (4) burnout prevalence, and (5) workforce retention across human and veterinary surgical specialisation in the United Kingdom.

## Materials and methods

Study design

We conducted a descriptive, cross-sectional, comparative analysis of secondary, aggregated, publicly available data. No primary or individual-level data were collected. In this context, the sample refers to the collection of institutional datasets, workforce surveys, and published reports drawn from the sources. The study examined reports and peer-reviewed literature released between July and August 2025.

Data sources

A structured, web-based search protocol was implemented to identify datasets relevant to postgraduate surgical training, workforce outcomes, and burnout. The authors searched the official websites of the RCVS, ECVS, General Medical Council (GMC), Intercollegiate Surgical Curriculum Programme (ISCP), Royal College of Surgeons of England (RCS England), British Medical Association (BMA), British Veterinary Association (BVA), and NHS Employers, between July and August 2025. Each site was queried using internal search tools and site indexes, with the terms training, curriculum, salary, burnout, workforce report, and continuing professional development (CPD). Documents were included if they contained quantitative data relating to at least one of the following domains: training duration, tuition or examination cost, salary structure, burnout or wellbeing metrics, or workforce retention. When multiple annual editions existed, the latest complete version available as of August 2025 was used. All retrieved reports were archived with version date and URL for traceability, and dataset characteristics (publication year, respondent population, sample size, and response rate, where reported) were documented.

To identify peer-reviewed studies addressing burnout, well-being, and workforce outcomes in medicine and veterinary practice, a structured search was performed across PubMed, Scopus, and Google Scholar, between July 1 and August 31, 2025. The following Boolean search strings were applied: (“veterinary” OR “veterinarian”) AND (“burnout” OR “wellbeing” OR “stress” OR “mental health”) AND (“United Kingdom” OR “UK”), and (“surgeon” OR “physician”) AND (“burnout” OR “wellbeing” OR “mental health”) AND (“United Kingdom” OR “UK”).

Studies were included if they: (1) involved UK-based clinical populations or internationally recognised reference surveys relevant to the UK workforce (e.g., Vet-SQ, Merck Animal Health, and BMA reports); (2) reported quantitative or mixed-methods data on burnout, wellbeing, or job satisfaction; and (3) were published in English between 2000 and 2025.

Exclusion criteria comprised non-clinical populations, intervention studies, or abstracts lacking full text. Where multiple overlapping publications existed, the most recent or most comprehensive version was selected. Reference lists of key studies were manually screened to identify additional eligible papers.

A comprehensive list of data sources, extracted variables, and literature sources is provided in Tables 1-2.

**Table 1:** Comprehensive List of Data Sources and Extracted Variables Source: [1-5,8,14-20]

Category	Source/Institution	Document/Report (Year)	Access Date (2025)	Key Extracted Variables	URL/DOI
Institutional/Regulatory	Intercollegiate Surgical Curriculum Programme (ISCP)	General Surgery Curriculum: Purpose (2021)	August	Training structure; phases; time to CCT	https://www.iscp.ac.uk/iscp/curriculum/general-surgery-curriculum/2-purpose/
Institutional/Regulatory	Royal College of Veterinary Surgeons (RCVS)	Veterinary Graduate Development Programme (VetGDP): Information for Graduates (2023)	August	Transition period duration; graduate support framework	https://www.rcvs.org.uk/lifelong-learning/veterinary-graduate-development-programme-vetgdp/information-for-graduates/
Institutional/Regulatory	European College of Veterinary Surgeons (ECVS)	How to Become a Specialist (2024)	August	Residency length; eligibility; exam structure; fees	https://www.ecvs.org/ckfinder/userfiles/files/Membership/Become%20a%20Diplomate/How%20to%20become%20a%20Specialist.pdf
Institutional/Regulatory	Royal College of Veterinary Surgeons (RCVS)	Continuing Professional Development (CPD) (2025)	August	CPD requirements; typical costs; hours per year	https://www.rcvs.org.uk/lifelong-learning/continuing-professional-development-cpd/
Institutional/Workforce	NHS Employers	Pay and Conditions Circular (Medical and Dental) 2/2025 (2025)	August	Salary bands (F1-Consultant); study leave provisions	https://www.nhsemployers.org/system/files/2025-06/Pay-and-Conditions-Circular-MD-2-2025_0.pdf
Institutional/Workforce	General Medical Council (GMC)	National Training Survey Results (2023)	August	Burnout risk prevalence; trainee wellbeing indicators	https://www.gmc-uk.org/-/media/documents/national-training-survey-2023-initial-findings-report_pdf-101939815.pdf
Institutional/Workforce	British Medical Association (BMA)	Consultant Workforce Survey Report (2019)	August	Consultant wellbeing; intent to retire; workload	https://www.bma.org.uk/media/3429/bma-consultant-workforce-shortages-and-solutions-oct-2020.pdf
Institutional/Workforce	British Medical Association (BMA)	Caring for the Mental Health of the Medical Workforce: The Evidence Base (2022)	August	Synthesis of mental health evidence; workforce risk factors	https://www.bma.org.uk/media/ckshvkzc/bma-mental-health-survey-report-september-2024.pdf
Institutional/Workforce	Society of Practising Veterinary Surgeons (SPVS)	Salaries and Benefits Survey (2025)	August	Median veterinary salaries by role; ranges	https://spvs.org.uk/2025-salary-survey/
Institutional/Workforce	Royal College of Veterinary Surgeons (RCVS)	RCVS Facts 2021: Annual Report (2022)	August	Workforce numbers; de-registrations; qualifications	https://www.rcvs.org.uk/news-and-views/publications/rcvs-facts-2021
Institutional/Workforce	British Veterinary Association (BVA)	Voice of the Veterinary Profession Survey (2019)	August	Anxiety/depression prevalence; workforce attitudes	https://www.rcvs.org.uk/news-and-views/publications/the-2019-survey-of-the-veterinary-profession/1the-2019-survey-of-the-veterinary-profession-report-final.pdf
Institutional/Workforce	Royal College of Physicians (RCP)	Census of Consultant Physicians and Higher Specialty Trainees in the UK 2020-21 (2022)	August	Consultant appointments; retention; workload	https://www.rcpe.ac.uk/sites/default/files/2021_consultant_census_report.pdf
Institutional/Workforce	Royal College of Surgeons of England (RCS England)	UK Surgical Workforce Census (2023)	August	Workforce projections; surgeon supply; attrition pressures	https://www.rcseng.ac.uk/standards-and-research/surgical-workforce-census/our-actions/#:~:text=Back%20to%20objectives-,Ensuring%20a%20strong%20and%20sustainable%20surgical%20workforce,set%20out%20in%20the%20charter

**Table 2:** Data Sources, Extracted Variables, and Literature Sources for Peer-Reviewed Data Source: [6,7,9-13]

Category	Source/Institution	Document/Report (Year)	Access Date (2025)	Key Extracted Variables	Sample Size/Respondent Group	URL/DOI
Peer‑Reviewed	Mudry et al.	Development and longitudinal validation of the Veterinary Stressors Questionnaire (Vet‑SQ) (2025)	August	Stress/burnout indices; validation; longitudinal follow‑up	n = 3,244 baseline; n = 674 follow‑up	https://doi.org/10.1038/s41598-025-05965-3
Peer‑Reviewed	Bartram et al.	Mental health and well‑being of UK veterinary professionals: a systematic review (2009)	August	Historical prevalence of anxiety, depression, and suicidal ideation	Systematic review (multiple studies)	https://doi.org/10.1007/s00127-009-0030-8
Peer‑Reviewed	Ashton-James and McNeilage	Stress and Wellbeing Factors Contributing to Burnout in a Specialist Small Animal Hospital (2022)	August	Work‑related stressors; qualitative/quantitative burnout determinants	Specialist referral hospital cohort	https://doi.org/10.3389/fvets.2022.942778
Peer‑Reviewed	West et al.	Physician burnout: contributors, consequences, and solutions (2018)	August	Medical burnout drivers; systems factors; interventions	Narrative review	https://doi.org/10.1111/joim.12752
Peer‑Reviewed	Patel et al.	Factors related to physician burnout and its consequences: a review (2018)	August	Correlates of physician burnout: consequences; mitigation	Review	https://doi.org/10.3390/bs8110098
Peer‑Reviewed	Hatch et al.	Workplace stress, mental health, and burnout of veterinarians in Australia (2011)	August	Veterinary burnout context; predictors; prevalence	Australian veterinarians (~500)	https://doi.org/10.1111/j.1751-0813.2011.00833.x
Peer‑Reviewed	Volk et al.	Merck Animal Health Veterinarian Wellbeing Study II - Executive Summary (2020)	August	US benchmark wellbeing indices; stress and satisfaction	US veterinarians (~2,800)	https://doi.org/10.2460/javma.256.11.1237

Inclusion and exclusion criteria

Data were included if they were (1) publicly available, (2) UK-based, and (3) explicitly addressed surgical training structure, financial cost, salary, retention, or wellbeing. Excluded sources comprised non-UK publications, non-surgical disciplines, private institutions without verifiable financial data, and reports lacking methodological transparency or numerical results. Small-animal surgery (veterinary) and general surgery (human) were selected as representative analogues, due to similar operative breadth and postgraduate duration.

Variables and bias

Six analytical domains were compared: (1) training duration, (2) financial cost, (3) salary trajectory, (4) burnout prevalence, (5) workforce retention, and (6) career sustainability indicators.

Cost calculation

The total estimated training cost for each profession was calculated by summing the following verified components: (1) undergraduate tuition over five years, (2) mandatory examination fees (MRCS (Member of the Royal College of Surgeons) + FRCS (Fellow of the Royal College of Surgeons) for human surgeons; ECVS/RCVS examinations for veterinary surgeons), and (3) self-funded CPD or specialist training courses, where these were explicitly stated in institutional guidance. For veterinary data, the average of published course fees (RCVS, AO Vet 2024-2025) was used to estimate CPD expenditure. For human surgical training, employer-funded courses were assumed to incur zero direct personal cost, in accordance with NHS Employers’ published pay and training frameworks. 

Potential biases included non-response bias in survey-based datasets (e.g., BVA and BMA surveys), reporting bias in employer-submitted pay data, and selection bias from limiting inclusion to publicly available UK reports.

Data extraction

Each report and publication was independently reviewed by two investigators. Extracted variables included publication year, dataset size (if available), outcome measures (e.g., mean salary and burnout prevalence), and methodological notes. Where overlapping or inconsistent data appeared, the most recent and methodologically transparent source was prioritised. When numeric ranges were reported (e.g., salary bands), the median value of the range was calculated to provide a single representative figure for descriptive comparison.

Data analysis

As this analysis used pre-aggregated and heterogeneous data from separate sources, inferential statistical testing was not performed and would not have been appropriate. The available data are presented descriptively. For financial variables presented as ranges, the median of the reported range was calculated for illustrative summary (e.g., £38,000-£51,000 reported as £44,500). Salaries are representative of figures available at the time. All values were expressed in 2024-2025 Great British Pounds.

Ethical statement

All the data were obtained from publicly available sources; no identifiable personal information was used, and ethical approval was not required.

Reporting statement

This study was conducted and reported in accordance with the STROBE (Strengthening the Reporting of Observational Studies in Epidemiology) guidelines for cross-sectional studies, and a completed checklist is provided in Appendix 1.

## Results

Training pathway and duration 

Data from the ISCP [1] and the RCVS [2] indicate that both pathways require more than a decade of structured postgraduate training. Veterinary surgeons complete the RCVS Veterinary Graduate Development Programme (VetGDP) [2], followed by one or two internships and a three-year ECVS/RCVS residency [3]. Human trainees complete Foundation, Core, and Higher Specialty Training phases under the ISCP curriculum [1]. The training pathway and duration is provided in Table 3.

**Table 3:** Training Pathway and Duration BVetMed - Bachelor of Veterinary Medicine; BVSc - Bachelor of Veterinary Science; VetGDP - Veterinary Graduate Development Programme; ECVS - European College of Veterinary Surgeons; RCVS - Royal College of Veterinary Surgeons; MBBS - Bachelor of Medicine, Bachelor of Surgery; MBChB - Bachelor of Medicine and Bachelor of Surgery; FRCS - Fellow of the Royal College of Surgeons; CCT - Certificate of Completion of Training Source: [1,2]

Profession	Undergraduate	Transition Period	Core/Internship Training	Higher/Specialist Training	Final Qualification	Total Duration
Veterinary surgeon	5 years BVetMed/BVSc	18 months VetGDP [2]	12 months rotating + 12 months surgical internship	3 years residency	ECVS/RCVS Specialist	11-12 years
Human surgeon	5 years MBBS/MBChB	2 years foundation	2 years core surgical training	6 years higher specialty training	FRCS + CCT [1]	15 years

Estimated financial cost of training 

Data from RCVS CPD guidance (2025) [4] and the NHS Employers Pay Circular (2/2025) [5] show that veterinary surgeons self-fund postgraduate education, including mandatory CPD and recommended AO Vet or arthroscopy courses [14]. NHS trainees receive funded study leave and examination support via nationally standardised pay structures [15]. The estimated financial cost of training is provided in Table 4.

**Table 4:** Estimated Financial Cost of Training CPD - Continuing Professional Development; ECVS - European College of Veterinary Surgeons; MRCS - Member of the Royal College of Surgeons; FRCS - Fellow of the Royal College of Surgeons; NHS - National Health Service Note: Costs are approximate and represent direct personal or employer-funded expenses reported for 2024-2025 Source: [4,5,14,15]

Cost Component	Veterinary (£)	Human (£)
Undergraduate tuition (5 y)	45,000	45,000
CPD and specialist courses	≈6,500	Employer-funded
Examination fees	900-1,300 (ECVS)	3,625 (MRCS + FRCS)
Total estimated cost	≈56,000	≈48,600

Salary trajectory

According to NHS Employers and the Society of Practising Veterinary Surgeons [5,20], salaries are indicative and vary by institution; these reflect published figures at the time of data collection (August 2025) [15]. Human surgical trainees are salaried and pensioned throughout training, whereas veterinary residents’ pay is typically lower than new-graduate starting salaries until specialist certification. The salary trajectory is provided in Table 5.

**Table 5:** Salary Trajectory F1 - Foundation year 1; F2 - Foundation year 2 Source: [5,15,20]

Stage	Veterinary (£)	Human (£)
New graduate/F1	32,000-37,100	38,000
Year 2/F2	34,000-40,500	44,000
Internship	19,600-37,100	-
Residency	38,000-51,000	52,000-74,000
Specialist/Consultant	70,000-100,000+	109,000-145,000

Burnout and wellbeing

Data from the GMC National Training Survey (2023) [4] show that 67,000 of 101,000 medical trainees (66%) reported moderate-to-high burnout risk. Among consultant surgeons, 9,000 of approximately 30,000 (30%) described poor or deteriorating wellbeing in the BMA Consultant Workforce Survey [5]. In the veterinary sector, 1,700 of 3,269 respondents (52%) reported anxiety or depression in national BVA surveys [18], while Vet-SQ longitudinal data [9] demonstrated persistently high stress indices (n = 3,244 baseline; n = 674 follow-up). Historical data from Bartram et al. [10] show 452 of 2,152 (21%) UK veterinarians reporting suicidal ideation and 560 (26%) reporting anxiety. Across both professions, source data consistently identified workload intensity, financial strain, and limited autonomy as key stressors, rather than making direct inter-professional comparisons. 

Retention and workforce sustainability

Data from the RCP and the RCS Eng show that approximately 4,000 of 5,700 (70%) surgical consultants remain in substantive NHS practice 10 years post-CCT [16,17], with 1,400-1,600 (20%-25%) transitioning to private or non-clinical roles. Among veterinary professionals, 540 of 4,500 (12%) reported an intention to leave practice within five years, and approximately 90 (20%) of those de-registrants held postgraduate qualifications [18,19]. Available longitudinal data specific to veterinary specialists remains limited but suggests higher stability than general practice.

Career sustainability indicators

Qualitative reviews highlighted three recurring challenges across both professions: (1) workload imbalance and scheduling pressures, especially in surgical and emergency settings; (2) financial disparity during training, particularly for veterinary interns and residents; and (3) limited institutional wellbeing frameworks, with only medicine having a structured national survey mechanism [6].

## Discussion

Our analysis provides a descriptive, policy-focused overview rather than a statistical, comparative study. This comparative analysis demonstrates clear structural and financial contrasts between human and veterinary surgical specialisation in the United Kingdom. Both professions require more than a decade of postgraduate training and substantial financial and personal investment, yet differ markedly in organisation, funding, and long-term sustainability.

There is limited empirical research directly comparing postgraduate surgical training across these two professions. Existing literature addresses wellbeing, training satisfaction, or workforce dynamics within each field individually, but no prior study has integrated these variables into a unified framework [4-13,16-20]. The absence of comparative evidence limits external validation, but underscores the originality and relevance of the present findings.

Human surgical training benefits from a nationally standardised and centrally funded model. The NHS provides structured progression through salaried training posts, defined competency milestones, and consistent regulatory oversight via the ISCP and GMC [1,15]. This framework offers predictable career advancement and financial stability throughout training. In contrast, veterinary surgical training remains decentralised and variably resourced. Internships and residencies often lack formal salary protection or funding parity, relying on employer goodwill or self-funding. This heterogeneity introduces financial barriers, extends the time to economic recovery, and increases the inequality of access to specialist qualifications [2,3,9].

The current findings align with the Mudry et al. (2025) study [9] and earlier work by Bartram et al. (2009) [10], Hatch et al. (2011) [12], and Ashton-James and McNeilage (2022) [11], all of which identify sustained occupational stress and moral strain across veterinary sectors. Comparable evidence exists in human medicine: West et al. (2018) [6] and Patel et al. (2018) [7] describe multifactorial burnout among physicians, while the BMA (2022) report [8] documents worsening mental health and early retirement intentions. While these datasets differ in structure and measurement tools, their collective trends consistently demonstrate high rates of psychological distress across both professions. 

Although inferential statistics were not applicable due to heterogeneity in reporting methods, descriptive trends reveal shared stressors: heavy workload, limited work-life balance, and delayed financial security. These findings should be interpreted as broad, policy-relevant observations rather than direct inter-professional comparisons. They highlight a broader issue of professional sustainability across both sectors.

Economic disparities compound these challenges. The SPVS (2025) salary survey [20] demonstrates that, while veterinary specialists may eventually approach consultant-level earnings, the path remains financially disadvantageous during training. Human trainees, though modestly paid, receive consistent income and funded educational support, allowing a more stable return on investment. These observations reflect differing structural mechanisms, rather than quantifiable financial “risk.”

From a policy perspective, structured veterinary residency funding aligned with NHS salary scales could improve access and retention. Establishing an RCVS-led wellbeing survey, akin to the GMC National Training Survey, would enable systematic monitoring and targeted intervention. Enhanced access to confidential, stigma-free mental-health support is vital for both professions. Conversely, human training could benefit from the veterinary model’s early operative exposure and integrated clinical-academic design.

Both systems demand prolonged commitment and technical excellence, yet operate under growing workforce strain. Sustainable training models, equitable funding, and coherent wellbeing strategies are crucial to maintaining a resilient surgical workforce.

Limitations

This study’s design and data structure have several important limitations. It relied exclusively on secondary, aggregated data from institutional reports and published literature, rather than individual-level datasets, which restricts reproducibility and statistical comparability. Data sources were heterogeneous in methodology, timing, and definitions. For example, some burnout measures were based on stress indices rather than validated psychological scales, and salary datasets reported differing cost components (e.g., inclusion or exclusion of benefits, bonuses, or overtime pay). As such, all comparisons are descriptive and non-inferential.

Potential selection bias exists because only publicly available and English-language UK sources were included. Unpublished, regional, or non-English data may have been omitted. Financial comparisons are further constrained by structural differences in funding models, whereby veterinary trainees predominantly incur personal and out-of-pocket costs. Medical trainees, on the other hand, operate within a mixed personal-employer-funded NHS system. Salary and cost figures are therefore approximate and may vary significantly by institution, region, and year. Variable definitions and incomplete metadata in certain reports (e.g., response rates or sampling frames) may also have introduced reporting bias. Training durations assume uninterrupted progression. These factors limit external validation and the precision of cross-sector comparisons.

Nevertheless, triangulating verified national datasets provides a transparent and policy-relevant, descriptive overview of current training and workforce dynamics in both professions.

## Conclusions

This descriptive analysis highlights structural and financial differences and similarities between human and veterinary surgical specialisation in the UK. They share extensive training requirements and similar professional challenges, but differ fundamentally in structure and financial support. Data from national regulatory and professional sources indicate that veterinary surgeons face greater personal financial investment and slower return on income, while human trainees benefit from salaried and centrally funded posts. Both groups demonstrate high burnout and retention pressures. The findings should be interpreted as descriptive trends that underscore areas for policy attention, rather than definitive comparison. Coordinated efforts to improve funding models, wellbeing monitoring, and cross-disciplinary learning could strengthen the sustainability of both surgical workforces.

## Appendices

Appendix 1

**Table 6:** STROBE Statement - Checklist of Items That Should Be Included in Reports of Observational Studies Strengthening the Reporting of Observational Studies in Epidemiology (STROBE, https://www.strobe-statement.org)

	Item No.	Recommendation	Page No.	Relevant Text From Manuscript
Title and abstract	1	(a) Indicate the study’s design with a commonly used term in the title or the abstract	N/a	Abstract: “Cross-sectional descriptive comparison”
		(b) Provide in the abstract an informative and balanced summary of what was done and what was found	N/a	Abstract: Background, Aim, Methods, Results, Conclusion
Introduction				
Background/rationale	2	Explain the scientific background and rationale for the investigation being reported	N/a	Introduction, paragraphs 1-3
Objectives	3	State specific objectives, including any prespecified hypotheses	N/a	Final paragraph of Introduction: “The primary objectives were to descriptively compare duration, cost, salary trajectory, burnout prevalence, and workforce retention…”
Methods				
Study design	4	Present key elements of study design early in the paper	N/a	Methods, “Study Design” - describes cross-sectional descriptive analysis of aggregated public data
Setting	5	Describe the setting, locations, and relevant dates, including periods of recruitment, exposure, follow-up, and data collection	N/a	Methods, “Study Design” and “Data Sources” (July-August 2025)
Participants	6	(a) Cohort study - Give the eligibility criteria, and the sources and methods of selection of participants. Describe methods of follow-up. Case-control study - Give the eligibility criteria, and the sources and methods of case ascertainment and control selection. Give the rationale for the choice of cases and controls. Cross-sectional study - Give the eligibility criteria, and the sources and methods of selection of participants	N/a	Methods, “Inclusion and Exclusion Criteria” (publicly available, UK-based, quantitative reports)
		(b) Cohort study - For matched studies, give matching criteria, and the number of exposed and unexposed. Case-control study - For matched studies, give matching criteria, and the number of controls per case	N/a	N/a
Variables	7	Clearly define all outcomes, exposures, predictors, potential confounders, and effect modifiers. Give diagnostic criteria, if applicable	N/a	Methods, “Variables and Bias” (six analytical domains)
Data sources/measurement	8	For each variable of interest, give sources of data and details of methods of assessment (measurement). Describe comparability of assessment methods if there is more than one group	N/a	Methods, “Data Extraction” and “Data Sources”; heterogeneity of veterinary vs. medical data noted (Table 1)
Bias	9	Describe any efforts to address potential sources of bias	N/a	Methods, “Variables and Bias” - describes non-response, reporting, and selection bias
Study size	10	Explain how the study size was arrived at	N/a	Not applicable - aggregated data from secondary institutional sources
Quantitative variables	11	Explain how quantitative variables were handled in the analyses. If applicable, describe which groupings were chosen and why	N/a	Methods, “Data Analysis” - medians used for ranges; all costs in 2024-2025 GBP
Statistical methods	12	(a) Describe all statistical methods, including those used to control for confounding	N/a	Methods, “Data Analysis” - inferential statistics not performed; descriptive summaries only
		(b) Describe any methods used to examine subgroups and interactions	N/a	N/a
		(c) Explain how missing data were addressed	N/a	N/a
		(d) Cohort study - If applicable, explain how loss to follow-up was addressed. Case-control study - If applicable, explain how matching of cases and controls was addressed. Cross-sectional study - If applicable, describe analytical methods, taking account of the sampling strategy	N/a	N/a
		(e) Describe any sensitivity analyses	N/a	N/a
Results				
Participants	13	(a) Report numbers of individuals at each stage of study—eg numbers potentially eligible, examined for eligibility, confirmed eligible, included in the study, completing follow-up, and analysed	N/a	Not applicable (aggregated institutional data)
		(b) Give reasons for non-participation at each stage	N/a	
		(c) Consider use of a flow diagram	N/a	
Descriptive data	14	(a) Give characteristics of study participants (eg demographic, clinical, social) and information on exposures and potential confounders	N/a	Results Tables 1-4 and describe dataset types, publication years, and populations
		(b) Indicate number of participants with missing data for each variable of interest	N/a	N/a, institutional reports
		(c) Cohort study - Summarise follow-up time (eg, average and total amount)	N/a	N/a
Outcome data	15	Cohort study - Report numbers of outcome events or summary measures over time	N/a	N/a
		Case-control study - Report numbers in each exposure category, or summary measures of exposure	N/a	N/a
		Cross-sectional study - Report numbers of outcome events or summary measures	N/a	Results, under “Burnout and Wellbeing” (numerators and denominators provided)
Main results	16	(a) Give unadjusted estimates and, if applicable, confounder-adjusted estimates and their precision (eg, 95% confidence interval). Make clear which confounders were adjusted for and why they were included	N/a	N/a
		(b) Report category boundaries when continuous variables were categorised	N/a	N/a
		(c) If relevant, consider translating estimates of relative risk into absolute risk for a meaningful time period	N/a	N/a
Other analyses	17	Report other analyses done - e.g., analyses of subgroups and interactions, and sensitivity analyses	N/a	N/a
Discussion				
Key results	18	Summarise key results with reference to study objectives	N/a	First paragraph of Discussion
Limitations	19	Discuss limitations of the study, taking into account sources of potential bias or imprecision. Discuss both direction and magnitude of any potential bias	N/a	Discussion, “Limitations” subsection
Interpretation	20	Give a cautious overall interpretation of results considering objectives, limitations, multiplicity of analyses, results from similar studies, and other relevant evidence	N/a	Discussion paragraphs 1-8; tempered language and comparison with prior literature
Generalisability	21	Discuss the generalisability (external validity) of the study results	N/a	Discussion, final paragraph (“These findings should be interpreted as descriptive trends…”)
Other information				
Funding	22	Give the source of funding and the role of the funders for the present study and, if applicable, for the original study on which the present article is based	N/a	None mentioned in disclosures
